# On the Principles Which Govern Treatment in Diseases and Disorders of the Heart

**Published:** 1900-06

**Authors:** 


**On the Principles which Govern Treatment in Diseases and Disorders of the Heart.** By Sir RICHARD DOUGLAS POWELL, Bart., M.D. Pp. 118. London: H. K. Lewis. 1899.

Perhaps the most interesting of these Lumleian lectures is the first, dealing with so-called functional diseases of the heart. The description of the neurotic temperament is lucid, and based upon discriminating observation. The knowledge of character of necessity plays an important part in the treatment of functional disease, as well as in diseases of more definite character, the symptoms of which may be aggravated by a neurotic disposition. The advice given for the management of such cases by Sir Douglas Powell contains the sound common-sense of a practical physician. In the two later lectures there is perhaps nothing worthy of special comment except the treatment of malignant endocarditis by injections of yeast-culture, and by nuclein. Of five cases treated with injections of yeast, one recovered. A table is given of the published cases of malignant endocarditis treated with anti-streptococcic serum up to the time of the lectures. In fourteen cases there were three recoveries.

